# Tackling the Challenges of Graft Healing After Anterior Cruciate Ligament Reconstruction—Thinking From the Endpoint

**DOI:** 10.3389/fbioe.2021.756930

**Published:** 2021-12-22

**Authors:** Shiyi Yao, Patrick Shu Hang Yung, Pauline Po Yee Lui

**Affiliations:** Department of Orthopaedics and Traumatology, The Chinese University of Hong Kong, Shatin, Hong Kong SAR, China

**Keywords:** ACL reconstruction, anterior cruciate ligament, ACL, graft healing, biological therapy, inflammation, osteogenesis, angiogenesis

## Abstract

Anterior cruciate ligament (ACL) tear is common in sports and accidents, and accounts for over 50% of all knee injuries. ACL reconstruction (ACLR) is commonly indicated to restore the knee stability, prevent anterior–posterior translation, and reduce the risk of developing post-traumatic osteoarthritis. However, the outcome of biological graft healing is not satisfactory with graft failure after ACLR. Tendon graft-to-bone tunnel healing and graft mid-substance remodeling are two key challenges of biological graft healing after ACLR. Mounting evidence supports excessive inflammation due to ACL injury and ACLR, and tendon graft-to-bone tunnel motion negatively influences these two key processes. To tackle the problem of biological graft healing, we believe that an inductive approach should be adopted, starting from the endpoint that we expected after ACLR, even though the results may not be achievable at present, followed by developing clinically practical strategies to achieve this ultimate goal. We believe that mineralization of tunnel graft and ligamentization of graft mid-substance to restore the ultrastructure and anatomy of the original ACL are the ultimate targets of ACLR. Hence, strategies that are osteoinductive, angiogenic, or anti-inflammatory should drive graft healing toward the targets. This paper reviews pre-clinical and clinical literature supporting this claim and the role of inflammation in negatively influencing graft healing. The practical considerations when developing a biological therapy to promote ACLR for future clinical translation are also discussed.

## 1 Introduction

### 1.1 Epidemiology of anterior cruciate ligament tears and current management

The anterior cruciate ligament (ACL) is a band of dense connective tissues that courses from the femur to the tibia, the function of which is to prevent excessive knee anterior–posterior translation and to maintain joint stability (Duthon et al., 2006; Zantop et al., 2006). Tears or ruptures of ACL are very common injuries in sports medicine, representing more than 50% of all knee injuries and affecting more than 200,000 people in the United States each year (Musahl and Karlsson, 2019).

ACL tears can impair knee function and increase the lifetime risk of knee osteoarthritis. Patients without and with meniscal tear had a 0%–13% and 21%–48% higher risk of developing knee osteoarthritis at 10 years after an ACL injury, respectively (Oiestad et al., 2009). The contribution of ACL tears to the burden of degenerative joint disease is substantial, accounting for an increase of 30,000–38,000 patients with symptomatic knee osteoarthritis and an additional 25,000–30,000 total knee arthroplasties each year in the United States. (Mather et al., 2013).

ACL tears are managed by conservative and surgical approaches. Cryotherapy, restrictive bracing, continuous passive motion, electrotherapy, and exercises aimed at reducing inflammation, restricting excessive knee motions, or strengthening muscles to improve knee symptoms and stability as well as to protect the joint are common conservative treatment options. However, conservative approaches for the management of ACL tears are poorly accepted by young active individuals. The continued active lifestyles of these individuals often lead to recurrent knee instability, chondral and meniscal injuries, and early onset of osteoarthritis (Raines et al., 2017).

ACL reconstruction is a surgical procedure that replaces the injured ACL with a tendon graft as the ACL does not heal after injury due to insufficient vascularization. Bone tunnels are artificially created in the distal femur and proximal tibia, and the tendon graft is inserted and fixed to the bone tunnels using staples, sutures, cross-pins, or interference screws. The procedure is usually assisted by arthroscopy to minimize the size of incision and reduce complications. The success rate of ACLR varied from 73% to 95% and the return to pre-injury activity level varied from 37% to 75% (Yunes et al., 2001; Fithian et al., 2005). Graft failure, presented as graft laxity and inferior mechanical properties compared with that of native ACL, is one of the main reasons for poor outcome after ACLR. A graft failure rate ranging from 1.5% to 5.7% has been reported (Yunes et al., 2001). Such graft failure is mainly attributed to surgical errors, traumatic injuries, failure of tendon graft-to-bone tunnel integration, and unsuccessful graft remodeling (George et al., 2006). Graft healing after ACLR is very slow, leading to long periods of carefully monitored rehabilitation and long delays before returning to full activity (Silva et al., 2012).

To tackle the problem of poor graft healing after ACLR, we believe a new research paradigm should be applied. An inductive approach, starting from the ideal endpoint of ACLR, should be adopted. The feasibility of translating the pre-clinical findings into clinical practice should be considered at the pre-clinical stage. This would enhance the future translation of the findings to benefit patients. Using this new paradigm of thinking, we believe that strategies that are osteoinductive, angiogenic, or anti-inflammatory should drive graft healing toward the ultimate goal of restoring ACL function after ACLR. This paper aims to present evidence from the literature to support this claim. The graft healing process is first summarized, with evidence supporting the role of inflammation in negatively influencing the healing response highlighted. Evidence supporting osteoinductive, angiogenic, or anti-inflammatory approaches to promote graft healing will then be systematically reviewed. Important issues requiring consideration when developing biological therapies for ACLR from the perspective of future clinical translation are then discussed.

## 2 Graft healing process after anterior cruciate ligament reconstruction

**FIGURE 1:**
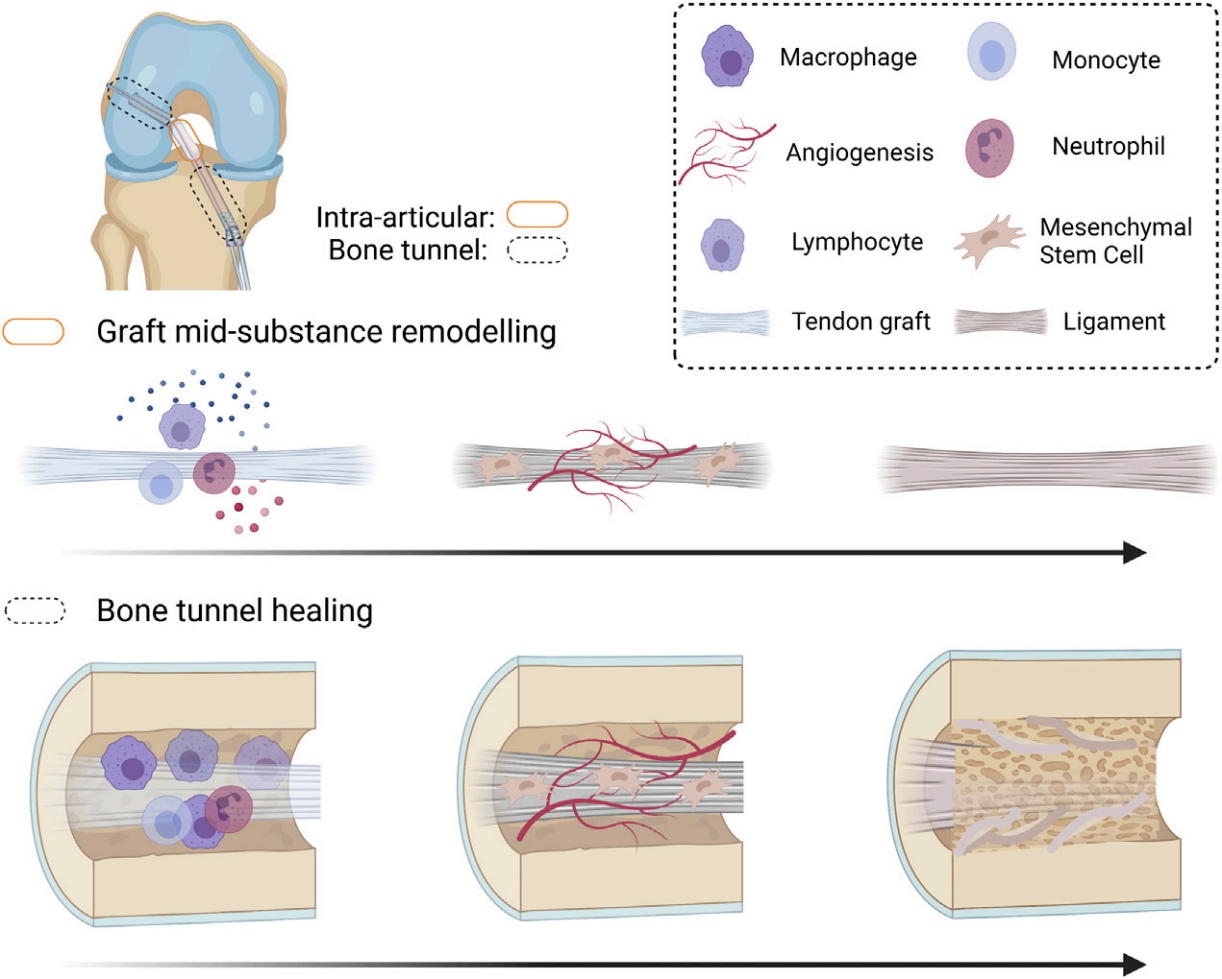
Schematic diagram summarizing the graft healing process after anterior cruciate ligament (ACL) reconstruction. After ACL reconstruction (ACLR), inflammatory occurs, attracting immune cells and mesenchymal stromal cells (MSCs) to the injured site. The original cells in the tendon graft undergo necrosis and are replaced by MSCs infiltrating into the graft. Both MSCs and the inflammatory cells produce angiogenic factors and the MSCs proliferate and differentiate. The differentiated MSCs produce extracellular matrix and remodeling enzymes to incorporate the tendon graft-to-bone tunnel by Sharpey’s fibers and is associated with improved biomechanical properties of the healing complex. However, there is regional variation in healing along the bone tunnel. The original ACL insertion site is not re-established. The tendon graft mid-substance theoretically should remodel to a ligament. However, it degenerates due to excessive inflammation and poor angiogenesis after ACLR. Created with BioRender.com.

Tendon graft-to-bone tunnel healing followed by graft mid-substance remodeling (collectively called “graft healing”) are two key healing processes that occur after ACLR (Figure 1). After ACLR, an inflammatory response is triggered, and inflammatory cells are recruited to the injury site. They clear cell debris and produce inflammatory cytokines, which attract mesenchymal stromal cells (MSCs) (formerly called mesenchymal stem cells) to the bone tunnel region and the intra-articular graft mid-substance. Next, cell necrosis in the implanted graft occurs. Re-vascularization and repopulation of tendon graft with MSCs then take place. The MSCs terminally differentiate and produce growth factors and extracellular matrix (ECM) to incorporate the tendon graft to the bone tunnel. On the other hand, with the help of the inflammatory cells and MSCs, the graft mid-substance remodels from a tendon to a ligament in a process called ligamentization (Scheffler et al., 2008).

### 2.1 Tendon graft-to-bone tunnel healing

Tendon graft-to-bone tunnel healing is the weak link in the early stage of ACLR as it requires the attachment of a compliant material-like tendon to a relatively stiff material-like bone. The regeneration of a normal insertion site with formation of a unique transitional tissue called “enthesis” (Petersen and Laprell, 2000), characterized by gradual change in structure, composition, and mechanical behavior, is pivotal for efficient transfer of load and prevention of stress accumulation at the interface. Excessive inflammation (Song et al., 2017) at the graft-to-bone tunnel interface induces the formation of a fibrous scar tissue interface rather than a normal insertion site. The mechanical strength achieved by this scar interface is far from satisfactory.

Depending on how the collagen fibers are attached to bone, there are two types of entheses at the bone–tendon junction—direct and indirect insertions. Direct insertion (also called the fibrocartilaginous enthesis) is composed of four zones (Petersen and Laprell, 2000) in order of gradual transition: tendon, uncalcified fibrocartilage, calcified fibrocartilage, and bone. Indirect insertion has no fibrocartilage interface. The tendon/ligament passes obliquely along the bone surface and inserts at an acute angle into the periosteum and is connected by Sharpey’s fibers over a broader area of tendon and bone. Direct and indirect insertions confer different anchorage strength and interface properties at the tendon–bone interface. Although both direct and indirect insertions have been described in the literature as a better healing outcome after ACLR (Liu et al., 1997), it has been more widely accepted that the insertion type after ACLR is an indirect one. Our results have shown that the chondrocytes at the tendon–bone tunnel interface only functioned as an intermediate in endochondral ossification and were replaced by bone with time during healing (Lui et al., 2010). The placement of tendon graft inside an artificially created bone tunnel, while providing a large bone surface area for tendon graft-to-bone tunnel healing, also disrupts the physiological mechanical load, resulting in regional-dependent stress shielding and subsequent bone loss (Lui P. P. Y. et al., 2013; Stolarz et al., 2017). The healing is, therefore, not uniform at different regions of bone tunnel and at different bone tunnels, with some areas exhibiting better healing than the others (Wen et al., 2010; Lui et al., 2015). Tunnel widening and bone resorption negatively influence healing and increase the chance of graft pull-out. Tendon to bone tunnel healing is slow, and the direct insertion site of native ACL is not regenerated. The outcome of graft healing after ACLR, therefore, remains unsatisfactory.

### 2.2 Graft mid-substance remodeling

As healing progresses, the weak link of graft healing gradually shifts from the tendon graft-to-bone tunnel interface to the graft mid-substance. The tendon graft undergoes “ligamentization,” in which the composition and organization of ECM are adapted to the functions of an active ACL.

Ligament fibroblasts have higher DNA content and are more metabolically active compared with tendon fibroblasts. Besides, the ECM of ligament contains more type III collagen and glycosaminoglycans but slightly less total collagen compared with tendons. Due to the direction of mechanical load, the collagen fibers of ligament are less orderly aligned compared with tendon. Moreover, the collagen fibrils in ligament are smaller but have more reducible cross-links compared with the fibrils in tendon (Amiel et al., 1984).

The process of ligamentization, therefore, requires the tendon graft to metabolically, compositionally, and ultrastructurally remodeled to adapt to the function of a ligament. However, the process is not successful as graft degeneration due to poor angiogenesis and ECM degradation were observed both clinically (Marumo et al., 2005) and in animal studies (Lui et al., 2014c; Yung et al., 2020).

## 3 Role of inflammation in influencing healing response

Shortly after graft implantation, an inflammatory response, which is important for removing cell debris and activate tissue repair, ensues. Neutrophils and macrophages are recruited to the tendon–bone interface as early as 4 days after surgery (Wall and Board, 2014). They produce inflammatory cytokines and fibrogenic growth factors, which contribute to the formation of a fibrous tissue interface between the graft and host bone at the early healing phase (Ekdahl et al., 2008). While the fibrous tissue fills the gap and provides early mechanical support at the interface, excessive inflammation causes fibrosis, which interferes with graft osteo-integration. Excessive inflammation also causes bone tunnel widening, peri-tunnel bone loss, and graft degeneration. Combining histological and molecular analyses, a clear correlation between increased expression of inflammatory cytokines, M1 macrophage infiltration, and graft loosening has been reported in tissue samples of patients who underwent a second-look arthroscopy within the first year after arthroscopic ACLR (Song et al., 2017).

To confirm the association of inflammation with poor outcome of ACL graft healing, a systematic search and analysis of the literature was done (search strategy in Supplementary Information S1). The results showed that excessive inflammation due to 1) ACL injury, 2) surgical trauma in ACLR, and 3) tendon graft-to-bone tunnel motion were common reasons for poor tendon graft-to-bone tunnel healing and graft mid-substance degeneration (Table 1). These unfavorable biological and mechanical factors induce the release of inflammatory cytokines by macrophages, synoviocytes, or fibroblasts, stimulating the production of matrix metalloproteinases (MMPs), which degrade ECM, and activating osteoclasts for bone resorption. The role of each of these factors in inducing excessive inflammation and impacting the outcomes of graft healing after ACLR are discussed below.

**TABLE 1:** A summary of the impact of inflammation on the outcomes of anterior cruciate ligament (ACL) graft healing.

Study type	Causes of inflammation^a^/cells and factors involved	Results	References
Human	(1)	IL-6 levels were significantly higher in the group with <6 weeks of injury than in the group with >12 weeks since injury. IL-6 was significantly elevated in painful ligamentous injury of knee, showed negative correlation with Lysholm knee scores at 2 months, 6 months, and 1 year of follow-up, and showed negative correlation with Tegner level of sports activity at 1 year of follow-up	Gupta et al. (2021)
	IL-6		
Human	(1)	High concentration of IL-6 and MMP-3 in the synovial fluid early post-ACL injury was associated with aberrant gait biomechanics in the injured limb at 6 months post-ACLR.	Evans-Pickett et al. (2021a)
	IL-6, MMP-3		
Human	(1)	At 2 years of follow-up, patients that failed to reach the QOL PASS threshold after surgery (*n* = 6, 27%) had significantly greater IL-1α, IL-1ra, MMP-9 concentrations on the day of surgery. Patients that failed to reach the IKDC PASS threshold (*n* = 9, 41%) had significantly greater IL-1α	Lattermann et al. (2018)
	IL-1α, IL-1ra, MMP-9		
Human	(1) (2)	Individuals with lesser biomechanical loading on the ACLR limb at the 6-month follow-up exam, compared with the contralateral limb, demonstrate greater concentrations of plasma MMP-3 and IL-6 early after ACL injury and during the early postoperative period	Pietrosimone et al. (2017)
	MMP-3, IL-6		
Human	(2)	Patients with Remnant Preserved (RP)-ACLR had better knee stability within 3 months which was associated with higher expression of IL-8 in the synovial fluid compared with the patients with conventional ACLR	Kim et al. (2020)
	IL-8		
Human	(2)	Graft loosening was closely related to increased gene and protein expression of inflammatory cytokines (TNF-α, IL-6, and IL-8) within the first year of ACLR. There was a probable role of M1 but not M2 macrophages in the pathological process leading to graft loosening	Song et al. (2017)
	IL-6, IL-8, TNF-α, M1 Macrophage		
Human	(2)	Increased level of IL-10, IL-1β, IL-6, IFN-γ in the synovial fluid at 3–4 days post-ACLR was associated with a prolonged recovery	Inoue et al. (2016)
	IL-10, IL-1β, IL-6, IFN-γ		
Human	(2)	There is an association between tibial bone tunnel enlargement and elevated synovial fluid concentrations of IL-1β concentrations postoperatively after ACLR. A lower expression of IL-1β in the synovial fluid after autologous conditioned serum (ACS) treatment was associated with reduced tunnel widening 6 months and 1 year after ACLR.	Darabos et al. (2011a)
	IL-1β		
Human	(2)	An elevated synovial fluid concentration of IL-6, TNF-α, and NO at 7 days after ACLR was associated with tibial bone tunnel enlargement at 38 ± 7 weeks after surgery	Zysk et al. (2004)
	TNF-α, IL-6, NO		
Rat	(2)	The peri-tunnel bone loss correlated with high expression of MMP1, MMP13, and CD68^+^ cells at the graft–bone tunnel interface at week 6 after ACLR.	Lui et al. (2015)
	MMP1, MMP13, CD68^+^ cells		
Rat	(2)	Alendronate reduced peri-tunnel bone resorption, increased mineralized tissue inside bone tunnel as well as histologically and biomechanically promoted graft-bone tunnel healing at week 6, probably by reducing the expression of MMP1, MMP13, and CD68-positive cells	Lui et al. (2013a)
	MMP1, MMP13, CD68^+^ cells		
Rat	(2)	Macrophage depletion following ACLR significantly improved histological and biomechanical properties of the healing tendon–bone interface at 42 days	Hays et al. (2008)
	Macrophages		
Rat	(3)	Short-duration of low-magnitude cyclic axial loading of the ACL graft was associated with more inflammatory ED1 macrophages and less bone formation in the bone tunnel at 5, 14, 28 days post-ACLR.	Brophy et al. (2011)
	ED1 macrophages		
Rat	(3)	Interface width was smaller and collagen fiber continuity was greater in the immobilized group. Immobilized animals exhibited fewer ED1 + macrophages at the healing interface at 2 and 4 weeks. In contrast, there were more ED2 + macrophages at the interface in the immobilized group at 2 weeks	Dagher et al. (2009)
	ED1macrophages		
	ED2 macrophages		
Mice	(3)	A short period of immobilization after ACLR enhanced graft-to-bone tunnel healing by mitigating excessive MMP expression at day 30	Nakagawa et al. (2019)
	MMP -2, -3, -9, -13		

Note. *N.B.*

a Causes of inflammation: (1) ACL injury; (2) surgical trauma in ACLR, and (3) tendon graft-to-bone tunnel motion.

### 3.1 Anterior cruciate ligament injury

Synovial fluid is an interstitial fluid produced by synoviocytes in the synovial membrane and functions to absorb shock and reduce friction as well as transport nutrient and waste in the joint. ACL injury has been demonstrated to increase the level of inflammatory cytokines and related substances in the synovial fluid, including TNF-α, IL-1β, IL-6, IL-8, IL-1ra, and IL-10 (Irie et al., 2003). The synovial fluid leaks into the bone tunnel and exposes the tendon graft to the pro-inflammatory cytokines and catabolic enzymes, contributing to bone resorption and poor graft healing. This is evident by Berg et al. (2001), who have reported that tunnel healing was slower and less complete in the articular part of the tunnel than in the tunnel part, which was farther from the synovial environment. Besides, the level of inflammatory cytokines (IL-1) in the synovial fluid taken intra-operatively was associated with higher pain score and anterior knee laxity as well as lower functional outcome and return to sports in patients undergoing ACLR at 1-year follow-up (Gupta et al., 2021). In addition, high concentration of IL-1 and MMP-3 in the synovial fluid early post-ACL injury was also associated with aberrant gait biomechanics in the injured limb at 6 months post-ACLR (Evans-Pickett et al., 2021b). In another clinical study, patients failing to meet the Knee Injury and Osteoarthritis Outcome Score (KOOS QoL) Patient Acceptable Symptom State (PASS) threshold and the International Knee Documentation Committee scores (IKDC) PASS threshold 2 years post-ACLR had significantly higher pre-operative concentrations of inflammatory cytokines and matrix remodeling enzyme in the synovial fluid (Lattermann et al., 2018). These studies support that the inflammatory cytokines in the synovial fluid after ACL tear negatively influences graft healing.

### 3.2 Surgical trauma in anterior cruciate ligament reconstruction

ACLR constitutes a second trauma to the acutely injured knee, resulting in further elevation of the concentrations of inflammatory cytokines in the synovial fluid (Larsson et al., 2017). In a secondary analysis of data from 14 patients in a clinical trial, ACLR triggered a second inflammatory hit in the knee with an increase in the levels of IL-1β and IL-6 in the synovial fluid (Hunt et al., 2021). In a second-look arthroscopy, there were higher expressions of pro-inflammatory cytokines and signaling mediators in the graft tissues in the graft loosening group compared with the expressions in the normal graft group. The activated M1 macrophages accumulated in the graft tissue, but no M2 macrophages were identified (Song et al., 2017). A significant negative correlation between the expressions of inflammatory cytokines and knee laxity function score was observed in the patient. The level of IL-1β in the synovial fluid at 3–4 days post-surgery predicted poor functional recovery at 3 months after ACLR (Inoue et al., 2016). In another clinical trial, all patients with tibial tunnel enlargement had elevated synovial levels of TNF-α, IL-6, and NO at 7 days post-operation (Zysk et al., 2004). These findings support that inflammation after ACLR negatively impacts graft healing.

The release of inflammatory cytokines attracts inflammatory cells to the injury sites. The inflammatory cells release inflammatory cytokines and stimulate further inflammation in a viscous cycle. Among the limited numbers of studies investigating the temporal changes of inflammatory cells after ACLR, neutrophils and macrophages accumulated sequentially at the healing graft-to-bone interface and repopulated the tendon graft. T-lymphocytes and mast cells were seen occasionally at the graft-to-bone interface (Kawamura et al., 2005). Unlike the allograft group, the ratio of CCR4^+^CCR6^+^ Th to Treg cells in the synovial fluid post-surgery did not correlate with anterior knee laxity after ACLR in the autograft group in a clinical study (Yang et al., 2012).

Macrophages play an important role in orchestrating graft healing after ACLR. In an animal study of the temporal changes of pro-inflammatory, phagocytic M1 macrophages and anti-inflammatory, regenerative M2 macrophages, the M1 macrophages appeared as early as 4 days after ACLR at the tendon-to-bone interface and led to the formation of a scar-tissue interface rather than the reformation of a normal insertion site. The M2 macrophages did not appear until day 11 after ACLR (Kawamura et al., 2005). Lui et al. (2015) have also reported that higher expressions of M1 macrophages, MMP1 and MMP13 at the peri-tunnel region were associated with greater peri-tunnel bone loss at the tibia after ACLR, and local administration of alendronate can reduce peri tunnel bone resorption, increase mineralized tissue inside bone tunnel probably by reducing the expression of MMP1, MMP13, and CD68-positive cells (Lui P. et al., 2013). Interestingly, the depletion of macrophages following ACLR significantly improved the histological and biomechanical properties of the healing tendon–bone interface (Hays et al., 2008) further supporting that macrophage-induced excessive inflammation caused poor graft healing after ACLR.

### 3.3 Tendon graft-to-bone tunnel motion

Besides, tendon graft motion inside the bone tunnel also induces an inflammatory response and is one of the mechanisms causing poor graft healing (Rodeo et al., 2006). It induces macrophage infiltration, increases inflammation, increases bone resorption, and impairs graft incorporation. In contrast, inhibition of early graft-to-bone tunnel motion reduces macrophage accumulation, reduces excessive inflammation, and promotes more effective graft incorporation. In this regard, graft healing in the femoral tunnel was inversely proportional to the magnitude of graft-tunnel motion. Daily low-magnitude cyclic axial load of ACL graft in the postoperative period induced greater infiltration of inflammatory macrophages and less bone formation in the bone tunnel compared with the immobilization group in a rat ACLR model (Brophy et al., 2011). On the contrary, there was better graft healing in the immobilization group compared with the graft healing in animals with normal cage activity. Graft failure at the intra-articular portion at a higher maximum load was observed in the immobilization group, whereas graft pullouts at a lower maximum load were observed in the normal cage activity group (Sakai et al., 2000). In another study, there were fewer M1 macrophages, more M2 macrophages at the tendon–bone interface, smaller interface width, higher collagen fiber continuity, higher failure load, and higher stiffness in the immobilization group compared with the free-cage activity group post-ACLR (Dagher et al., 2009). Therefore, suppressing inflammation by stable graft fixation and early healing with reduced tendon graft-to-bone tunnel motion are both crucial to the success of ACLR.

However, immobilization alone does not provide all the appropriate signals to improve healing. Rather, delayed and controlled mechanical loading after an initial period of immobilization is needed. This is evident by the greater suppression of MMP activities at the graft-to-bone interface and better graft healing in animals immobilized for 5 days or 14 days post-ACLR compared with the MMP activities and graft healing in the animals without immobilization or prolonged immobilization (Nakagawa et al., 2019). In another study, there were significantly fewer inflammatory M1 macrophages and significantly more resident M2 macrophages at the healing graft-to-bone interface in the day-4 and -10 delayed-loading groups compared with the counts in the immediate-loading and prolonged immobilization groups at 2 and 4 weeks after ACLR. There were less scar tissue and more bone formation at the interface as well as higher mechanical strength of the reconstructed ACL complex in the delayed mobilization groups compared with the immediate-loading group and prolonged immobilization group (Packer et al., 2014). Tight control of the mechanical environment at the graft-to-bone tunnel interface is hence, crucial to the maintenance of an anti-inflammatory and regenerative environment favorable for graft healing.

## 4 An inductive approach for developing new biologics for improving graft healing

To tackle the problem of biological graft healing, we believe that an inductive approach should be adopted, starting from the optimal endpoint after ACLR, and even the results may not be achievable at present, followed by developing clinically practical strategies to achieve this ultimate target. This would help in refining the scope of the clinical problem and directing us to test for the right strategies.

The motivation for ACLR is to replace the damaged ligament with the tendon graft and transform it into a ligament. Ideally, the tunnel graft should mineralize and completely be replaced by bone with re-establishment of a fibrocartilage transition zone at the original footprints of ACL. Both angiogenesis and inflammation are known to regulate tissue repair. Consequently, we believe that strategies that can enhance osteogenesis at the bone tunnel, promote angiogenesis, and suppress inflammation at the knee and bone tunnels would promote graft healing after ACLR. To support our claim, a systematic review of literature examining the efficacy of biological therapies in the promotion of graft healing was performed (search strategy in Supplementary Information S2). The results showed that most (41/49, 83.7%) of the biologics with positive effects on graft healing have either osteogenic, angiogenic, or anti-inflammatory properties (Table 2).

**TABLE 2:** A summary of the nature and effects of biologics on graft healing after anterior cruciate ligament reconstruction (ACLR).

	Osteogenesis	Angiogenesis	Suppression of inflammation	Other mechanisms	Outcomes of graft healing (+/-/no effect)	Remarks	References
Animal Studies							
ADSC sheet	V				+	ADSCs stimulate bone-forming activities. ADSC sheets improved biomechanical strength, prevented bone tunnel enlargement, and promoted tendon–bone interface healing and graft remodeling in ACLR	Matsumoto et al. (2021)
BMSCs	V				+	BMSCs stimulate bone formation. It promoted graft osteointegration at the tendon–bone interface after ACLR	Hur et al. (2019)
BMP-2 Binding Peptides	V				+	The incorporation of BMP-2 binding peptides into materials used for ACLR enhanced bone formation and healing inside bone tunnels	Crispim et al. (2018)
BMSCs transfected with TGF-β gene	V				+	BMSCs are stem cells with osteogenic differentiation capacity. TGF-β is an osteogenic growth factor. BMSCs overexpressing TGF-β promoted tendon-to-bone healing after ACLR by upregulating the TGF-β/MAPK signaling pathway	Wang et al. (2017)
PRP + BMSCs	V				+	BMSC has osteogenic differentiation potential and PRP can stimulate this potential. PRP significantly stimulated osteogenic differentiation of BMSCs. The combination of PRP and BMSCs enhanced bone formation, maturation of graft-to-bone tunnel interface, and biomechanical properties of the bone–graft–bone complex	Teng et al. (2016)
SHMSP	V				+	SHMSP is an osteogenic factor. It enhanced tunnel bone formation after ACLR	Al-Bluwi et al. (2016)
AdTGF-β₁	V				+	TGF-β is an osteogenic growth factor. Hamstring tendon transfected with AdTGF-β₁ gene promoted healing of tendon–bone interface after ACLR	Wang et al. (2015b)
hUCB-MSCs	V				+	hUCB-MSCs enhanced tendon-bone healing through broad fibrocartilage formation with higher histological scores and decreased femoral and tibial tunnel widening compared with the control group	Jang et al. (2015)
BMSC infected with BMP-2 gene	V				+	BMSCs have osteogenic differentiation potential. BMP-2 can induce osteogenic and chondrogenic differentiation of pluripotent stem cells and bone progenitor cells. The transplantation of BMSCs genetically modified with BMP-2 enhanced the osseointegration of the tendon graft within the host bone	Dong et al. (2012)
Rat kidney cell line transduced with pCMV-BMP-2 gene	V				+	BMP-2 is an osteogenic growth factor. It enhanced osteogenesis at the tendon graft-to-bone tunnel interface after ACLR.	Wang et al. (2010)
PRP/DPB complex	V				+	The mixture of PRP/DPB enhanced chondrogenesis, Sharpey’s fiber formation and graft incorporation into the bone tunnel at 60% PRP	Zhao and Zhai, (2010)
TGF-β1	V				+	TGF-β1 is an osteogenic growth factor. It enhanced tunnel bone formation	Yamazaki et al. (2005)
TGF-β+EGF	V				+	TGF-ß increases both collagen and noncollagenous protein synthesis. EGF stimulates fibroblast proliferation *in vitro*. Application of TGF-β and EGF improved the structural properties of the bone–graft–bone complex after ACLR	Yasuda et al. (2004)
Bone-derived extract (Bone Protein, Sulzer Biologics, Wheat Ridge, Colorado)	V				+	Bone-derived extract (Bone Protein, Sulzer Biologics, Wheat Ridge, Colorado) is effective in augmenting bone ingrowth. It improved healing of a tendon graft in a bone tunnel in an intra-articular ligament-reconstruction model	Anderson et al. (2001)
ACL-derived CD34^+^ cell sheet transduced with VEGF gene or sFLT-1		V			+	ACL-derived CD34^+^ cells expressing moderate levels of VEGF improved tendon graft maturation and biomechanical strength; however, CD34^+^ overexpressing VEGF promoting excessive angiogenesis impeded graft healing and mechanical strength. The transplantation ACL-derived CD34^+^ cell sheet secreting sFLT1, a soluble VEGF inhibitor decreased angiogenesis, delayed graft maturation, and decreased biomechanical strength of the bone–graft–bone complex	Takayama et al. (2015)
VEGF		V			_	VEGF is an angiogenic growth factor. Excessive angiogenesis reduced integrity and stiffness as well as increased laxity of graft	Yoshikawa et al. (2006)
PDGF-BB		V			+	PDGF has a positive effect on revascularization. The local long-term application of PDGF using a biodegradable drug delivery tool biomechanically and histologically improved free tendon graft remodeling after ACLR	Weiler et al. (2004)
Synovium-derived cells pre-treated with TGF-β1 or TGF-β1			V		+	The transplantation of synovium-derived cells cultured in TGF-β1 or TGF-β1 inhibited the deterioration of the intra-articular part of tendon graft after ACLR	Kondo et al. (2011)
PRP	V	V			+	PRP contains PDGF, VEGF, and TGF-β. It increased the bioactivity of the tendon–bone interface and resulted in histological improvement at the tendon–bone junction	Zhang et al. (2019)
hBMSC-CM	V	V			+	hBMSC-CM contains a variety of growth factors, including TGF-β, VEGF, and IGF. It accelerated graft osteo-integration and mid-substance ligamentization after ACLR	Sun et al. (2019)
Muscle-Secreted Factors	V	V			+	Muscle-secreted factors influences revascularization and tendon–bone closure. Using a rat model of ACLR showed that conditioned media derived from human muscle tissue accelerated femoral tunnel closure, a key step for autograft integration	Ghebes et al. (2018)
α-FGF	V	V			+	α-FGF is a mitogenic factor of osteoblasts and chondrocytes as well as an angiogenic factor. It induced fibrocartilage formation at the tendon–bone interface after ACLR	Lu et al. (2018)
ACL-derived CD34^+^ cells transduced with BMP-2	V	V			+	ACL-derived CD34^+^ cells transduced with BMP-2 can stimulate angiogenesis and osteogenesis at the graft-bone interface. ACL-derived CD34^+^ cells transduced with BMP-2 accelerated graft–bone integration after ACLR	Kawakami et al. (2017)
BMSCs and VEGF	V	V			+	BMSCs have osteogenic potential and VEGF promotes angiogenesis. All parameters using MRI, collagen type III expression, and biomechanical analysis of pullout strength of the graft showed that application of intra tunnel BM-MSCs and VEGF enhanced tendon-to-bone healing after ACLR	Setiawati et al. (2017)
BMSCs genetically modified with bFGF/BMP-2	V	V			+	bFGF can promote angiogenesis and BMP-2 has osteogenic potential. The addition of BMP-2 or bFGF by gene transfer resulted in better cellularity, new bone formation, and higher mechanical property, which contributed to the healing process after ACLR	Chen et al. (2016)
ADRC	V	V			+	ADRCs secrete significantly larger amounts of growth factors, such as VEGF, hepatocyte growth factor than BMSCs. Local administration of ADRCs promoted the early healing process at the tendon–bone junction, both histologically and mechanically, after ACLR	Kosaka et al. (2016)
Fibrin clot	V	V			+	Transplantation of fibrin clot improved graft healing as shown by histology and MRI	Hensler et al. (2015)
TDSC sheet	V	V			+	TDSCs are stem cells with osteogenic differentiation capacity. TDSC sheet expressed bFGF, TGF-β1 and BMP-2 which have angiogenic and osteogenic effects. The transplantation of TDSC sheet promoted bone formation, enhanced graft osteointegration and graft mid-substance integrity, as well as improved biomechanical properties of the bone–graft–bone complex	Lui et al. (2014c)
TGF-β1 plasmid in liposomes	V	V			+	TGF-β1 increases angiogenesis and induces fibroblast, monocyte, and macrophage migration to sites of injury, promoting ligament healing. Injection of TGF-β1 plasmid in liposomes into bone tunnel improved biomechanical characteristics of the bone–graft–bone complex	Qin et al. (2013)
ACL-derived CD34^+^ cell sheet	V	V			+	CD34^+^ cells are endothelial cells that secrete angiogenic and osteogenic factors. The transplantation of ACL-derived CD34^+^ cell sheet enhanced healing of the bone–tendon junction and the grafted tendon by promoting proprioceptive recovery, graft maturation, and biomechanical strength. The outcomes were better after transplantation of the cell sheet compared with cell injection	Mifune et al. (2013)
ACL-derived CD34^+^ cell	V	V			+	CD34^+^ cells are endothelial cells which secrete angiogenic and osteogenic factors. Intracapsular injection of CD34^+^ cells post-ACLR increased biomechanical strength of the bone–graft–bone complex *via* enhancement of angiogenesis and osteogenesis at graft-bone interface at early stage after ACLR	Mifune et al. (2012)
Platelet	V	V			+	Platelet contains PDGF, VEGF, and TGF-β. The addition of blood platelets resulted in significant reduction in anterior-posterior knee laxity after ACLR	Spindler et al. (2009)
G-CSF	V	V			+	G-CSF contributes to angiogenesis and osteogenesis. A local application of G-CSF-incorporated gelatin significantly accelerated bone-tendon interface strength *via* enhanced angiogenesis and osteogenesis	Sasaki et al. (2008)
Human Studies							
hUCB-MSCs	V				No effect	The transplantation of allogeneic hUCB-MSCs did not show any clinical advantage such as the prevention of tunnel enlargement, knee laxity, and clinical outcomes	Moon et al. (2021)
PRP	V				+	PRP contained bone growth factors. The administration of PRP decreased the rate of second ACL injury compared with the literature	Berdis et al. (2019)
PRP	V				No effect	The administration of PRP did not prevent tunnel enlargement after ACLR	Sözkesen et al. (2018)
PRP	V				+	PRP contained bone growth factors. The application of PRP prevented femoral tunnel widening in ACLR	Starantzis et al. (2014)
PRP	V				No effect	The application of PRP did not reduce tunnel enlargement after ACLR	Vadalà et al. (2013)
PRP	V				+	PRP contained bone growth factors. It enhanced the formation of focal areas of sclerotic cortical bone with subsequent fusion into a thick tibial tunnel wall after ACLR	Rupreht et al. (2013b)
PRP	V				No effect	The administration of PRP to bone tunnels reduced tunnel widening, but the difference was not statistically significant	Mirzatolooei et al. (2013)
PRP	V				No effect	PRP contained growth factors with osteogenic activities. There was no significant improvement in tendon graft incorporation to the bone tunnel after ACLR	Silva and Sampaio, (2009)
PRPG		V			+	PRPG contains PDGF. It decreased edema and increased vascularity at the tibial tunnel after ACLR	Rupreht et al. (2013a)
PG		V			+	PG contained PDGF. It enhanced vascularization at the tibial tunnel interface and intra-articular part of the graft	Vogrin et al. (2010)
PRF	V	V			No effect	The administration of PRF did not significantly improve graft failure and graft ligamentization up to 12 months post-ACLR	Zeman et al. (2018)
PRP	V	V			No effect	The administration of PRP did not accelerate graft interface healing and graft ligamentization after ACLR	Komzák et al. (2015)
PRFM	V	V			+	PRFM has a substantial amount of growth factors (such as TGF-β1, PDGF, VEGF). PRFM-augmented patients showed a statistically significant higher patient-reported knee function	Del Torto et al. (2015)
PRP	V	V			+	The administration of PRP accelerated graft mid-substance remodeling after ACLR.	Seijas et al. (2013)
PRP	V	V			+	PRP contained PDGF, TGF-β1, and VEGF which were osteogenic and angiogenic. The administration of PRP enhanced graft mid-substance remodeling compared with the untreated grafts	Sánchez et al. (2010)
ACS	V		V		+	ACS contains endogenous anti-inflammatory cytokines including IL-1Ra and growth factors (IGF-1, PDGF, and TGF-b1) in the liquid blood phase. Intra-articular administration of ACS decreased bone tunnel widening and reduced the level of IL-1β in the synovial fluid after ACLR	Darabos et al. (2011b)

Note. N.B.

ACS, autologous conditioned serum; ADSC, adipose derived stem cell; ADRC, adipose-derived regenerative cell; AdTGF-β1, adenovirus-mediated transforming growth factor β1; BMP-2, bone morphogenetic protein-2; BMSCs, bone marrow mesenchymal stem cells; hBMSC-CM, hBMSC–conditioned medium; pCMV, plasmid cytomegalo virus; DPB, deproteinized bone; EGF, epidermal growth factor; α-FGF, acidic fibroblast growth factor; bFGF, basic fibroblast growth factor; G-CSF, granulocyte colony-stimulating factor; HGF, hepatocyte growth factor; IGF-1, insulin-like growth factor-1; PDGF-BB, platelet-derived growth factor–BB; PG, platelet gel; PRPG, platelet-rich plasma gel; PRFM, platelet-rich fibrin matrix; PRF, platelet-rich fibrin; PRP, platelet-rich plasma; SHMSP, Sadat–Habdan mesenchymal stimulating peptide; TGF-β, transforming growth factor–β; TDSC, tendon-derived stem cell; hUCB-MSCs, umbilical cord blood-derived mesenchymal stem cells; VEGF, vascular endothelial growth factor.

### 4.1 Osteogenesis

As indicated, the tunnel graft should ideally be mineralized, and the fibrocartilage transition zone should be re-established at the original ACL footprints. The importance of bone formation at the early stage of healing is demonstrated by the positive correlation of the mechanical strength of bone-tendon junction with the amount of osseous ingrowth, mineralization, and maturation of healing tissue (Sun et al., 2019; Zhang et al., 2019). Our previous study has shown replacement of tendon graft by bone at some regions at the femoral tunnel and juxta-articular segment of the tibial tunnel in a rabbit ACLR model, and we believe that this may represent the ideal healing inside the bone tunnel (Lui et al., 2010).

In preclinical studies, osteoinductive factors, such as bone morphogenetic protein-2 (BMP-2) (Crispim et al., 2018), α-fibroblast growth factor (α-FGF) (Lu et al., 2018), basic fibroblast growth factor (bFGF) (Kimura et al., 2008), granulocyte colony-stimulating factor (G-CSF) (Sasaki et al., 2008), and transforming growth factor-β1 (TGF-β1) (Yamazaki et al., 2005) enhanced tunnel bone formation and, hence, promoted tendon graft-to-bone tunnel healing after ACLR (Table 2). Besides, cells like ACL-derived CD34^+^ cell (Mifune et al., 2012; Mifune et al., 2013), tendon-derived stem cells (TDSCs) (Lui et al., 2014c), bone marrow-derived MSCs (BMSCs) (Hur et al., 2019), and adipose-derived stem cells (ADSCs) (Matsumoto et al., 2021) had been applied directly or made into cell sheets for the enhancement of bone formation after ACLR. These cells could release osteogenic factors and promote osteogenesis at the graft–bone interface. To enable the stable delivery and controlled release of osteogenic factors, both BMP-2 and TGF-β have been overexpressed in cells (Wang et al., 2010; Dong et al., 2012; Wang et al., 2017) to lengthen the therapeutic window and maximize their effect on bone formation. There was better tendon-to-bone integration and biomechanical properties of the bone–graft–bone complex in these groups compared with those in the control groups after ACLR. In consideration of the potential safety and difficulty in clinical application, weekly intra-articular injection of BMSC-conditioned medium (Sun et al., 2019) containing osteoinductive factors has been developed and is shown to promote tendon graft-to-bone tunnel integration and ligamentization after ACLR.

Clinically, platelet-rich plasma (PRP) containing osteogenic factors is frequently tested for the promotion of graft healing after ACLR with mixed results. It has been reported to prevent tunnel widening (Starantzis et al., 2014) and reduces the rate of revision of ACL surgery (Berdis et al., 2019). However, it has no significant effect on tunnel enlargement (Mirzatolooei et al., 2013; Vadalà et al., 2013) and graft incorporation (Silva and Sampaio, 2009) in other studies. This may be due to differences in study design (randomized controlled trials versus case series), PRP preparation, surgical procedures (single-bundle versus double-bundle and graft fixation methods), patient characteristics (adolescents versus adults), and rehabilitation protocols.

In summary, all pre-clinical studies have shown positive effects of osteoinductive factors on the promotion of bone formation and enhancement of tendon graft-to-bone tunnel healing, whereas the graft healing effect of PRP, which contain a complex mixture of bioactive proteins other than osteoinductive factors, remains controversial. More well-controlled studies are needed to confirm the effect of PRP on osteogenesis and graft healing after ACLR.

### 4.2 Angiogenesis

Adequate revascularization is critical for successful tendon graft-to-bone tunnel healing, by transporting MSCs and growth factors to the injury site. The infiltrated MSCs repopulate the tendon graft, release growth factors, produce collagen, and differentiated into cells of different lineages (bone, interface, or ligament) (Menetrey et al., 2008). Vascular endothelial growth factor (VEGF) has been shown to increase transiently, followed by blood vessel formation in the graft mid-substance at the early phase after ACLR in a rabbit model, suggesting that angiogenesis is involved in the early stage of graft mid-substance remodeling (Yoshikawa et al., 2006). On the other hand, low VEGF expression and vascularization were observed in tendon graft samples harvested in a patient with graft loosening in the second-look arthroscopy within the first year (Song et al., 2017), supporting that angiogenesis is important for successful graft mid-substance remodeling, in addition to tendon graft-to-bone tunnel healing.

The administration of angiogenic factors such as platelet-derived growth factor-BB (PDGF-BB) (Weiler et al., 2004), VEGF (Setiawati et al., 2017), and α-fibroblast growth factor (α-FGF) (Lu et al., 2018) has been reported to promote graft healing in different animal models of ACLR (Table 2). Furthermore, the application of cellular biologics in different forms, such as TDSCs (Lui et al., 2014c), ACL-derived CD34^+^ cell sheet (Mifune et al., 2013), or BMSC-conditioned medium (Sun et al., 2019) containing angiogenic factors, also promoted angiogenesis and accelerated graft healing after ACLR. However, excessive angiogenesis has also been reported to negatively impact the integrity of tendon graft, decrease the biomechanics of the reconstructed ACL, and increase the graft laxity (Yoshikawa et al., 2006). Tight control of the treatment dose and time of angiogenic factors is, hence, required.

There has been a long-term interest in the application of PRP and its related-blood preparations for the promotion of graft healing due to its angiogenic components (Sánchez et al., 2010). The administration of PRP enhanced graft mid-substance remodeling in some clinical studies (Seijas et al., 2013; Zeman et al., 2018), supporting that angiogenesis might be the plausible mechanisms underlying their action. However, there are also studies reporting that PRP was ineffective in the promotion of graft remodeling after ACLR (Komzák et al., 2015).

### 4.3 Suppression of inflammation

As discussed, inflammatory cytokines and fibrogenic growth factors contribute to the formation of a fibrous tissue interface between the graft and the bone tunnel at the early healing phase (Ekdahl et al., 2008). While the fibrous interface provides early mechanical support, it interferes with graft incorporation. Moreover, excessive inflammation causes bone tunnel widening, peri-tunnel bone loss, and graft degeneration. A correlation between M1 macrophage infiltration and graft loosening has been reported, while the depletion of inflammatory macrophages (Hays et al., 2008) promoted tendon graft-to-bone tunnel healing. In addition, intra-articular injection of alpha-2 macroglobulin, an MMP inhibitor, was reported to inhibit the enzymatic degradation of ACL after rupture in a rabbit model (Demirag et al., 2004). The collagen network was maintained, and the number of fibroblasts and fibrocytes was reduced in the ruptured ACL in the treatment group compared with that in the control group, supporting that suppression of inflammation can enhance graft integrity, which is crucial for providing mechanical strength of the ACL complex.

In our systematic search for biological interventions, only one preclinical study and one clinical study evaluating the efficacy of anti-inflammatory therapies could be identified. TGF-β1 is an anti-inflammatory growth factor. Kondo et al. transplanted TGF-β1-pretreated synovium-derived cells or TGF-β1 in a fibrin sealant sheet to a sheep ACLR model and found that both the pre-treated cells and TGF-β1 inhibited the deterioration of the intra-articular part of tendon graft with increased maximum load and stiffness of the bone–graft–bone complex at 12 weeks after reconstruction. Similar results were not observed after the transplantation of untreated cells in fibrin sealant sheet, suggesting that TGF-β1-inhibited graft degeneration is crucial for graft healing (Kondo et al., 2011), showing anti-inflammation effect. In a clinical study, intra-articular application of autologous conditioned serum (ACS) containing endogenous anti-inflammatory cytokines, including IL1Ra decreased bone tunnel widening after ACLR (Darabos et al., 2011a). The level of IL-1β in the synovial fluid of patients in the ACS group was significantly lower compared with that in the placebo group at early stage of ACLR, supporting that biological intervention, which inhibits intra-articular inflammation, can improve graft healing.

Despite the existence of clear evidence demonstrating the association of inflammation with poor healing outcomes (*Role of inflammation in influencing healing response* section), there has been relatively few original studies targeting inflammation in graft healing after ACLR compared with the number of studies investigating biological therapies targeting osteogenesis and angiogenesis. Improved understanding of the molecular mechanisms of inflammatory cells and cytokines in graft healing after ACLR would support the identification of new anti-inflammatory therapeutics.

## 5 Practical considerations of developing biologics for anterior cruciate ligament reconstruction—bring it closer to clinical application

While pre-clinical studies have shown positive effects of various biological therapies on graft healing, additional factors need to be considered for translating them into clinical practices. We suggested considering the following factors when developing new biologics at the early pre-clinical stage for generating clinically viable treatment strategies.

### 5.1 Compatibility with arthroscopy

Arthroscopy is a minimally invasive surgical procedure on a joint in which an arthroscope is inserted into the joint through a small incision to assist in knee examination and surgery. It is a common orthopedic procedure, and about 60,000 arthroscopic surgeries are performed annually in England only (Jameson et al., 2011). For arthroscopy-assisted ACLR, only two small incisions are made, one for the arthroscope and the other for the surgical instruments to be used in the knee cavity. This reduces recovery time due to trauma of the connective tissue. This is different from animal models of ACLR wherein an open surgery is usually performed. As arthroscopy-assisted ACLR is becoming the standard in clinical practice, and new interventions for improving graft healing must be compatible with arthroscopy. Strategies about how to deliver the biologic through the small incision in the presence of large volume of fluid during arthroscopy need to be developed for the clinical application of the biological therapy.

### 5.2 Localized and sustained intervention

As tendon graft-to-bone tunnel healing and graft mid-substance remodeling are complex healing processes with different endpoints (Ekdahl et al., 2008), localized and sustained interventions should be considered when developing new interventions. A carrier system that can localize the biologic to a specific site and supports its slow release is desired (Han et al., 2019). Collagen (Lu et al., 2018) and fibrin sealant (Kondo et al., 2011) were the carrier systems commonly used to deliver the biologics. Further studies on innovative carriers that can regulate the release of biologics consistent with the healing processes are needed.

### 5.3 Quality control

Various sources of cells have been used for ACLR (Wang et al., 2010; Wang et al., 2017; Hur et al., 2019). Issues related to the sources, purity, stability, storage condition, efficacy, safety, and delivery should be considered for clinical translation. MSC-based therapy is generally reported to be safe except in a few early reports wherein ectopic bone and tumor were observed in animal studies (Wang et al., 2015a; Zhong et al., 2017). Our previous study has shown that TDSCs are immune-privileged cells and might be used for allogeneic transplantation. Target TDSCs did not activate the immune system after immunization (Lui et al., 2014b). A low infiltration of T cells, M1 macrophages, M2 macrophages, and mast cells in the window wound was obtained after allogeneic TDSC transplantation to the tendon window wound (Lui et al., 2014a). A clinical-grade Good Manufacturing Practice-compliant (cGMP) stem cell bank and cell preparation protocol should be established for clinical application of stem cells for the augmentation of graft healing after ACLR.

PRP is another complex biological product that has been widely studied for the promotion of graft healing in ACLR both in preclinical and clinical studies with mixed results (Sözkesen et al., 2018; Berdis et al., 2019). There are different PRP preparations with different components including leukocyte-rich PRP, leukocyte-poor PRP, and platelet-rich plasma preparation rich in growth factors (PRGF) (Sánchez et al., 2010; Le et al., 2018). The standardization of PRP preparation with defined growth factor content is needed for its clinical translation.

**FIGURE 2:**
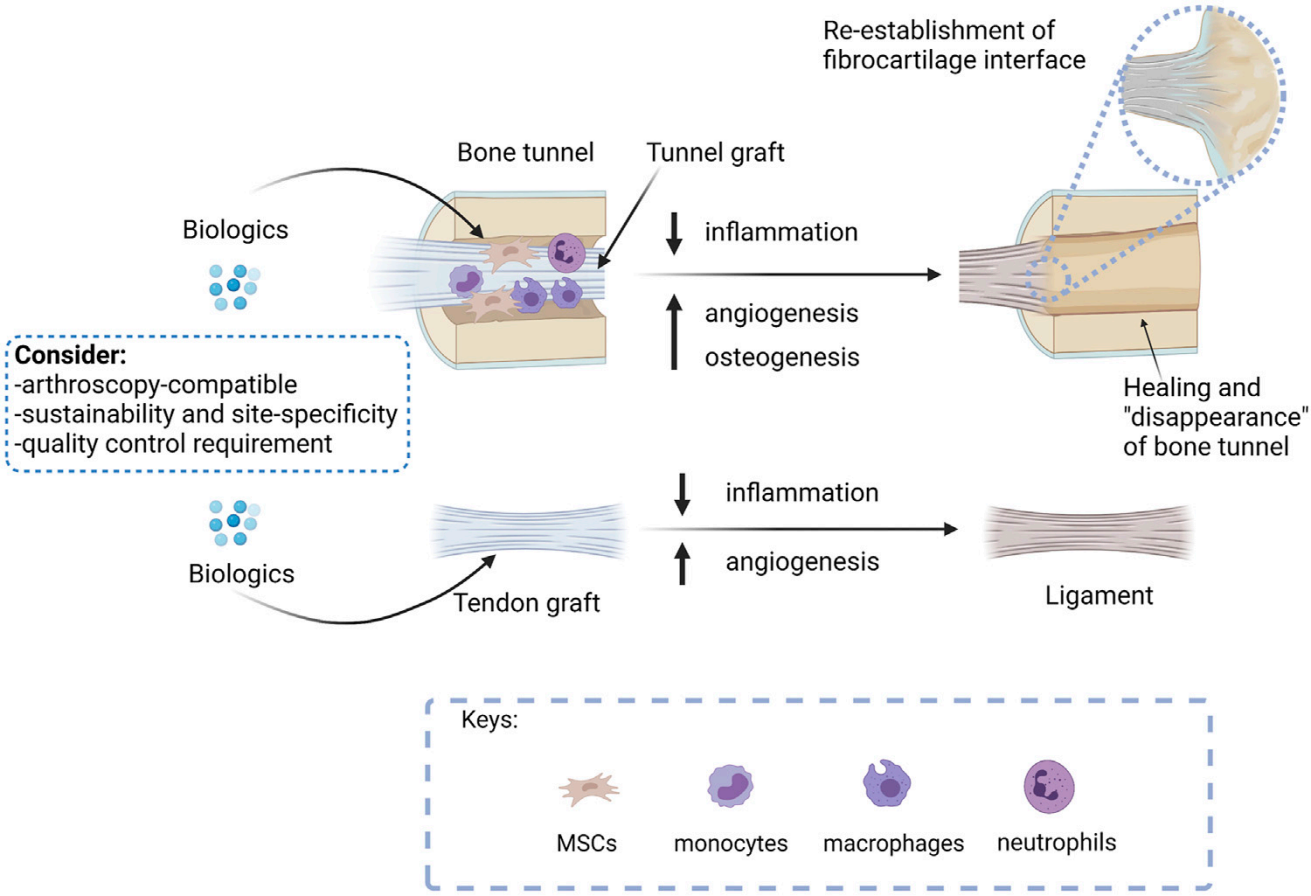
A summary of the treatment approaches and considerations for developing a clinically viable biological therapy for the promotion of graft healing after ACLR. The ultimate goal of ACLR is to replace the tunnel graft by bone with re-establishment of direct insertion at the original ACL footprint and remodel the tendon graft mid-substances to a ligament to meet the functions of an ACL. Biologics that promote bone healing, therefore, should promote tunnel healing, while biologics that enhance angiogenesis are expected to accelerate both tunnel healing and graft remodeling. As inflammation hampers graft healing, biologics that suppress inflammation are anticipated to promote both tunnel healing and graft remodeling. Researches developing osteogenic, angiogenic, or anti-inflammatory biologicals should consider if the proposed intervention is arthroscopy compatible, supports sustainable and site-specific application, and meets quality control requirements for successful future clinical translation. Created with BioRender.com.

Except pure proteins, biological therapies are usually complex products. In order to ensure their safety and efficacy in graft healing after ACLR, quality control is required. Figure 2 summarizes the treatment approaches and considerations for developing a clinically viable biological therapy for the promotion of graft healing after ACLR.

## 6 Conclusion

The adoption of an inductive approach in the research and development of new biologics for improving graft healing after ACLR with the consideration of the feasibility of clinical translation would help in refining the scope of the clinical problem and directing the search for the right strategies.

The bone tunnels in ACLR are artificially created. The ultimate targets after ACLR are to replace the tunnel graft with bone with the re-establishment of a direct insertion at the original ACL footprints and remodeling of graft mid-substance to a functional ACL. However, the remodeling of graft mid-substance always fails due to its avascularity. In addition, there is amble evidence showing that excessive inflammation due to an increase in inflammatory cytokines in the synovial fluid after ACL injury, surgical trauma in ACLR, and graft-to-bone tunnel motion because of unstable fixation and slow healing contribute to tunnel widening, bone resorption, and graft mid-substance degeneration. Therefore, strategies that can promote osteogenesis, angiogenesis, and anti-inflammation have a good chance of enhancing graft healing. This claim is supported by the detrimental effects of excessive inflammation on the healing outcomes, and the osteogenic, angiogenetic, or anti-inflammatory properties of most of the biologics that showed positive effects on graft healing in a systematic literature search. To enhance the translational potential of a new biologic for graft healing, its compatibility with the standard ACL surgical procedures, such as arthroscopy, its localized and sustained release pattern, and its quality control, are key issues to be considered as early as at the pre-clinical stage.

## Supplementary Material

Supplementary Information 1 Search strategy for articles examining the association of inflammation with poor outcome of ACL graft healing.

Supplementary Information 2 Search strategy for articles examining the efficacy of biological therapies in the promotion of graft healing.
