# Supplementary material for: Tackling the Challenges of Graft Healing After Anterior Cruciate Ligament Reconstruction—Thinking From the Endpoint
Source: Front Bioeng Biotechnol. 2021 Dec 22;9:756930. doi: 10.3389/fbioe.2021.756930 (PMC8727521; doi:10.3389/fbioe.2021.756930)
Supplement: Supplementary file 1 [file DataSheet2.docx]

**Supplementary information 2.** Search strategy for articles examining the efficacy of biological therapies in the promotion of graft healing.

A systematic search of the PubMed database using the keywords (ACLR OR ACL reconstruction OR anterior cruciate ligament reconstruction) AND (growth factor OR PRP OR cell) was done on 15^th^ Oct 2021.

The following inclusion and exclusion criteria are adopted.

Inclusion criteria:

1. Original human / animal studies of ACLR using a free tendon graft; and
2. Studies on the effectiveness of biological therapies including growth factor, cell on graft healing, graft laxity, knee stability or knee function after ACLR.

Exclusion criteria:

1. *In vitro* studies, *ex vivo* studies, cadaveric studies; review articles, technical notes, editorial comments;
2. Studies on ACLR using an allograft;
3. Studies not assessing graft healing after ACLR (e.g., post-ACLR osteoarthritis); and
4. Studies examining the effects of non-biological interventions including chemicals, biophysical interventions, implants, metal ions or biomaterial scaffold-only interventions

The search resulted in 373 studies. 63 original articles were identified after a preliminary screening of the article titles and abstracts and 49 articles were selected after a more detailed read of the articles.
