# Supplementary material for: Tackling the Challenges of Graft Healing After Anterior Cruciate Ligament Reconstruction—Thinking From the Endpoint
Source: Front Bioeng Biotechnol. 2021 Dec 22;9:756930. doi: 10.3389/fbioe.2021.756930 (PMC8727521; doi:10.3389/fbioe.2021.756930)
Supplement: Supplementary file 2 [file DataSheet1.docx]

**Supplementary information 1.** Search strategy for articles examining the association of inflammation with poor outcome of ACL graft healing.

A systematic search of the PubMed database with the keywords (“anterior cruciate ligament reconstruction” OR “ACL reconstruction” OR “ACLR”) AND (“inflammation” OR “inflammatory” OR “immune” OR “macrophage” OR “T-cell” OR “B-cell” OR “neutrophil” OR “lymphocyte” OR “mast cell” OR “interleukin” OR “TNF” OR “IFN” OR “MMP” OR “TIMP” OR “growth factor” OR “cytokine”) was done on 15^th^ Oct 2021. The search resulted in 271 studies. 29 original articles were identified after a preliminary screening of the article titles and 15 articles were selected after a more detailed read of the articles.

The following inclusion and exclusion criteria are adopted.

Inclusion criteria:

1. original human / animal studies of anterior cruciate ligament reconstruction (ACLR) using a free tendon graft; and
2. studies reporting the association of inflammation, inflammatory cytokines or inflammatory cells at the knee joint with outcomes related to graft healing, graft laxity, knee stability, knee function after ACLR.

Exclusion criteria:

1. *In vitro* studies, ex *vivo* studies, review articles, technical notes, editorial comments;
2. Studies not on ACLR;
3. Studies on ACLR using an allograft;
4. Studies without assessment on outcomes related to graft healing, graft laxity, knee stability or knee function after ACLR (e.g., post-ACLR osteoarthritis); and
5. Studies reporting the temporal changes of inflammatory cytokines or inflammatory cells only without reporting their associations with outcomes related to graft healing, graft laxity, knee stability or knee function after ACLR.
